# Urinary biomarkers of exposure to insecticides, herbicides, and one insect repellent among pregnant women in Puerto Rico

**DOI:** 10.1186/1476-069X-13-97

**Published:** 2014-11-19

**Authors:** Ryan C Lewis, David E Cantonwine, Liza V Anzalota Del Toro, Antonia M Calafat, Liza Valentin-Blasini, Mark D Davis, Samuel E Baker, Akram N Alshawabkeh, José F Cordero, John D Meeker

**Affiliations:** Department of Environmental Health Sciences, University of Michigan School of Public Health, 1415 Washington Heights, Ann Arbor, MI 48109 USA; Department of Obstetrics and Gynecology, Brigham and Women’s Hospital, 75 Francis Street, Boston, MA 02115 USA; University of Puerto Rico Graduate School of Public Health, Medical Sciences Campus, San Juan, 00935 Puerto Rico; National Center for Environmental Health, Division of Laboratory Sciences, Centers for Disease Control and Prevention, 4770 Buford Highway, Atlanta, 30341 GA USA; College of Engineering, Northeastern University, 360 Huntington Avenue, Boston, MA 02115 USA

**Keywords:** Biomarker, Pesticides, Pregnancy, Urine, Women

## Abstract

**Background:**

There are potential adverse health risks to the mother and fetus from exposure to pesticides. Thus, studies of exposure to pesticides among pregnant women are of interest as they will assist with understanding the potential burden of exposure globally, identifying sources of exposure, and designing epidemiology studies.

**Methods:**

We measured urinary concentrations of the insect repellent N-N-diethyl-meta-toluamide (DEET) and two of its metabolites [3-diethyl-carbamoyl benzoic acid (DCBA) and N,N-diethyl-3-hydroxymethylbenzamide (DHMB)], four pyrethroid insecticide metabolites [4-fluoro-3-phenoxybenzoic acid (4-F-3-PBA); 3-phenoxybenzoic acid (3-PBA); *trans*-3-(2,2-dichlorovinyl)-2,2-dimethylcyclopropane carboxylic acid (*trans*-DCCA); and *cis*-3-(2,2-dibromovinyl)-2,2-dimethylcyclopropane carboxylic acid (*cis*-DBCA)], and two chlorophenoxy herbicides [2,4-dichlorophenoxyacetic acid (2,4-D) and 2,4,5-trichlorophenoxyacetic acid (2,4,5-T)] in 54 pregnant women from Puerto Rico at three separate time points (20 ± 2 weeks, 24 ± 2 weeks, and 28 ± 2 weeks of gestation). We calculated the distributions of the biomarker concentrations and compared them to those of women of reproductive age from the general U.S. population where available, and estimated the within-subject temporal variability of these repeated measurements. We also collected questionnaire data on demographics, consumption of select fruits, vegetables, and legumes in the past 48-hr, and pest-related issues, and associations between these variables and biomarker concentrations were examined.

**Results:**

We found that 95th percentile urinary concentrations of DEET, 3-PBA, *trans*-DCCA, and 2,4-D were lower than women of reproductive age on the U.S. mainland, whereas 95th percentile urinary concentrations of 4-F-3-PBA, *cis*-DBCA, and 2,4,5-T were similar. DCBA, the only urinary biomarker detected in >50% of the samples, showed fair to good reproducibility across pregnancy (intraclass correlation coefficient: 0.60). Women were more likely (*p* <0.05) to have greater urinary concentrations of pesticide biomarkers if they were less educated (DCBA and *trans*-DCCA), unemployed (DHMB), or married (2,4-D), had consumed collards or spinach in past 48-hr (2,4-D) or had been using insect repellent since becoming pregnant (DCBA), or were involved with residential applications of pesticides (*trans*-DCCA).

**Conclusions:**

We identified concentrations and predictors of several pesticides among pregnant women in Puerto Rico. Further research is needed to understand what aspects of the predictors identified lead to greater exposure, and whether exposure during pregnancy is associated with adverse health.

## Background

Pesticides refer to a broad class of chemicals that prevent, destroy, repel, or mitigate any pests [1], such as unwanted insects (insecticides, insect repellents), weeds (herbicides), microbes (fungicides, disinfectants), and mice and rats (rodenticides) [2]. Due to their widespread use, exposures to pesticides have been documented in humans globally. Pesticides such as the insect repellent N-N-diethyl-meta-toluamide (DEET), the herbicides 2,4-dichlorophenoxyacetic acid (2,4-D) and 2,4,5-trichlorophenoxyacetic acid (2,4,5-T), and the metabolites of various pyrethroid insecticides (e.g., permethrin) have been measured in the urine of children [3–8] and adults [9, 10], including pregnant women [11–13]. These pesticides have also been measured in umbilical cord blood and sera [14, 15] and meconium [16] of newborn humans, and the breast milk of lactating humans [17, 18] and other mammals [19, 20], which suggests that fetal and early post-natal exposure to these pesticides is possible.

In the general population, skin contact is the primary route of exposure to DEET (e.g., applied directly to skin or clothing or bedding that makes contact with skin) [21], whereas exposure to both pyrethroids and chlorophenoxy acids occurs mainly through ingestion of tainted crops [20, 22]. However, depending on the scenario and population (e.g., occupational settings, improper product use), exposure to all of these pesticides may occur via several routes from a number of sources [20–22]. Epidemiology studies have linked exposures to pyrethroids or chlorophenoxy acids with lymphatic or blood cancers [6, 23, 24], and several adverse endocrine-related conditions, such as poor semen quality [25–28] and altered serum hormone levels [29]. In comparison, evidence of the impact of DEET on human health is limited and mostly derived from illness surveys and case reports [21]. Animal studies involving inhalation or oral exposures to DEET have demonstrated neurotoxicity of varying severity depending on the circumstances surrounding the exposures [21].

Despite the potential adverse health risks to both the mother and developing fetus, exposure studies in pregnant women have been limited for DEET, pyrethroids, and chlorophenoxy acids [11–13, 30–32]. Thus, additional exposure studies are necessary, which will in turn assist with understanding the potential burden of exposure globally, identifying sources of exposure, and designing epidemiology studies. Pregnant women living in Puerto Rico are one at-risk and important population to study as Puerto Rico has an unexplained increase in adverse pregnancy outcomes such as preterm birth over the past several decades [33] and the island has a history of pesticide drift associated with agricultural operations [34], pesticide contaminated hazardous waste sites [35], use of illegal pesticides [36], and use of pesticides in illegal ways (e.g., applying non-approved pesticides to crops) [37]. The primary aims of this study were to: 1) describe distributions, 2) assess within-subject temporal variability, and 3) identify predictors of urinary concentrations of DEET and two of its metabolites, four metabolites of synthetic pyrethroids, and two chlorophenoxy acids in a cohort of pregnant women from Puerto Rico. To our knowledge, this is the first biomarker study to report on exposures to select pesticides among pregnant women in Puerto Rico.

## Methods

### Study participants

This analysis concerned 54 pregnant women participating in the Puerto Rico Test site for Exploring Contamination Threats (PROTECT) project. PROTECT is an ongoing prospective birth cohort in the northern karst region of Puerto Rico designed to assess the potential relationship between environmental toxicant exposures and risk of preterm birth and other adverse pregnancy outcomes [38, 39]. Participants were recruited at approximately 14 ± 2 weeks of gestation at seven prenatal clinics and hospitals during 2010–2012. Pregnant women were eligible if they were 18–40 years of age, resided in a municipality within the northern karst region, received their first prenatal visit by the 20th week of pregnancy, did not use oral contraceptives three months prior to pregnancy or had in vitro fertilization as a method of assisted reproductive technology, and were free of known medical/obstetrics complications. Participants provided spot urine samples during three study visits at approximately 20 ± 2 weeks, 24 ± 2 weeks, and 28 ± 2 weeks of gestation. Questionnaires were also administered at each visit prior to collecting the urine to obtain information on demographics and self-reported consumption of fruits, vegetables, and legumes in the past 48-hr, and home pest-related issues. We also had questionnaire information on the use of insect repellent in the form sprays, lotions, or towelettes since becoming pregnant, which was collected from participants during visit 2 only. The study was described in detail to all participants who then gave informed consent. The Ethics and Research Committees of the University of Puerto Rico, the University of Michigan, and Northeastern University approved the research protocol. The involvement of the Centers for Disease Control and Prevention (CDC) did not constitute engagement in human subject research.

### Urinary biomarkers of pesticide exposure

**Figure 1:**
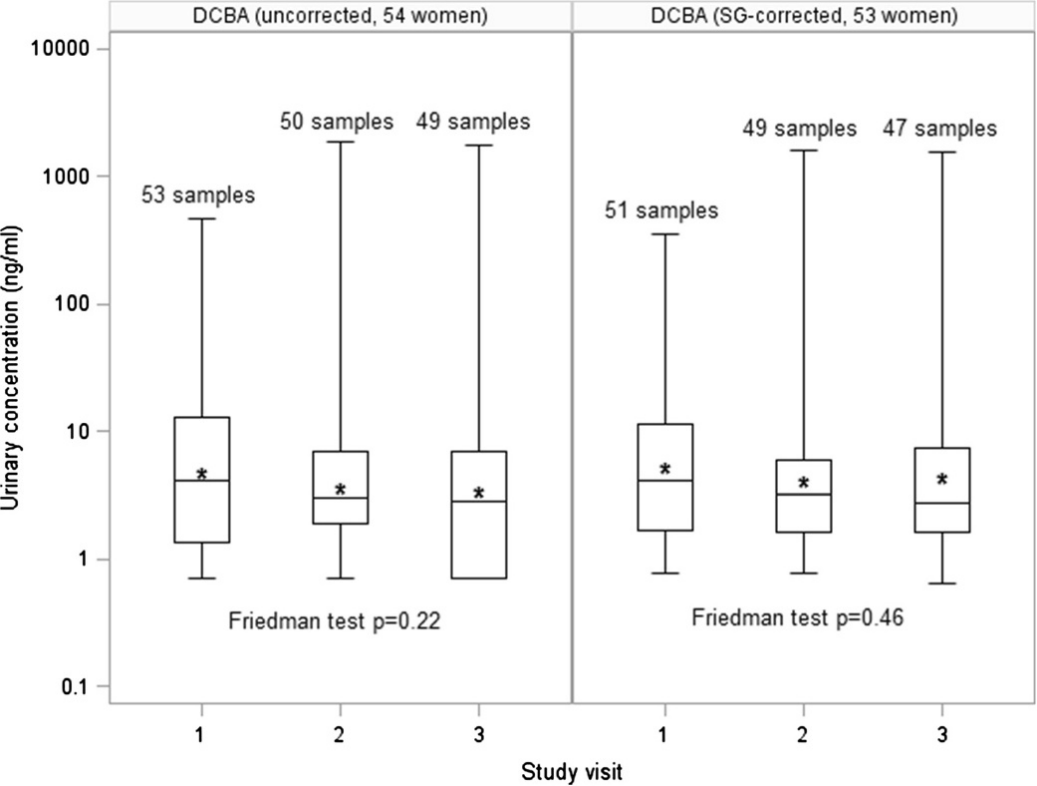
**Distributions of urinary concentrations of DCBA (ng/ml) in pregnant women between study visits (20 ± 2 weeks of gestation = visit 1, 24 ± 2 weeks of gestation = visit 2, and 28 ± 2 weeks of gestation = visit 3).** Boxes represent the interquartile range; horizontal lines represent the minimum, median, and maximum; and asterisks represent the geometric mean. Sample sizes differ between uncorrected and SG-corrected analyses because SG was not available for some samples.

At each study visit, participants provided one spot urine sample, which was collected and processed using procedures that were comparable to those the CDC has developed for the National Health and Nutrition Examination Survey (NHANES) and other studies. Urine samples were analyzed at the National Center for Environmental Health of the CDC (Atlanta, GA, USA) for the following 9 biomarkers: DEET and two of its metabolites, 3-diethyl-carbamoyl benzoic acid (DCBA) and N,N-diethyl-3-hydroxymethylbenzamide (DHMB); four metabolites of synthetic pyrethroids, 4-fluoro-3-phenoxybenzoic acid (4-F-3-PBA), 3-phenoxybenzoic acid (3-PBA), *trans*-3-(2,2-dichlorovinyl)-2,2-dimethylcyclopropane carboxylic acid (*trans*-DCCA), and *cis*-3-(2,2-dibromovinyl)-2,2-dimethylcyclopropane carboxylic acid (*cis*-DBCA); and two chlorophenoxy herbicides, 2,4-D and 2,4,5-T. The urinalysis used solid phase extraction and high-performance liquid chromatography-isotope dilution tandem mass spectrometry as described previously [40, 41]. Accuracy and precision for each analytical run were monitored through the use of calibration standards, reagent blanks, and quality control materials of high and low concentrations. For analyses concerning imputation of left-censored urinary concentrations (Table 1, Figure 1, and DCBA analyses in Tables 2, 3 and 4), concentrations below the limit of detection (LOD) were assigned a value of LOD divided by the square root of 2. Where adjustment for urinary output was necessary (Figure 1 and DCBA analyses in Tables 2, 3 and 4), urinary concentrations were corrected for specific gravity (SG), which was measured at the University of Puerto Rico using a digital handheld refractometer (Atago Co., Ltd., Tokyo, Japan), using the following formula: *P*_*c*_ = *P*_*m*_[(*SG*_*p*_ – 1)/(*SG*_*m*_ – 1)], where *P*_*c*_ is the SG-corrected urinary concentration (ng/ml), *P*_*m*_ is the measured urinary concentration (ng/ml), *SG*_*p*_ is the median of the urinary SGs for the population (1.019), and *SG*_*m*_ is the measured urinary SG.

**Table 1:** **Urinary concentrations of pesticide biomarkers (ng/ml, uncorrected for SG) in pregnant women from PROTECT (Puerto Rico) and comparison with women ages 18–40 from NHANES (U.S. population-based sample)**

								Percentiles				
Chemical class	Parent compound(s)	Analyte	LOD	Study and year	N	N (%) ≥ LOD	GM	25th	50th	75th	95th	Max
Insect repellent	DEET	DEET	0.1	PROTECT 10-12	152^a^	5 (3.3)	<LOD	<LOD	<LOD	<LOD	<LOD	3.3
			0.1	NHANES 01-02	456	67 (14.7)	<LOD	<LOD	<LOD	<LOD	0.2	57.3
		DCBA	1.0	PROTECT 10-12	152^a^	120 (79.0)	4.1	1.3	3.5	9.0	59.0	1856
		DHMB	0.1	PROTECT 10-12	152^a^	36 (23.7)	<LOD	<LOD	<LOD	<LOD	0.5	32.8
Pyrethroid insecticide	Cyfluthrin	4-F-3-PBA	0.1	PROTECT 10-12	116^b^	0 (0.0)	<LOD	<LOD	<LOD	<LOD	<LOD	<LOD
			0.1	NHANES 07-08	364	21 (5.8)	<LOD	<LOD	<LOD	<LOD	<LOD	4.6
	Cyhalothrin, Cypermethrin, Deltamethrin, Fenpropathrin, Permethrin, Tralomethrin	3-PBA	0.1	PROTECT 10-12	141^c^	65 (46.1)	0.2	<LOD	<LOD	0.6	2.3	11.3
			0.1	NHANES 07-08	339	235 (69.3)	0.4	<LOD	0.4	1.1	6.5	36.8
	Cypermethrin, Cyfluthrin, Permethrin	*trans*-DCCA	0.6	PROTECT 10-12	152^a^	11 (7.2)	<LOD	<LOD	<LOD	<LOD	2.4	7.6
			0.6	NHANES 07-08	362	54 (14.9)	<LOD	<LOD	<LOD	<LOD	3.3	29.0
	Deltamethrin	*cis-*DBCA	0.5	PROTECT 10-12	152^a^	0 (0.0)	<LOD	<LOD	<LOD	<LOD	<LOD	<LOD
			0.5	NHANES 07-08	364	5 (1.4)	<LOD	<LOD	<LOD	<LOD	<LOD	1.8
Chlorophenoxy herbicide	2,4-D	2,4-D	0.4	PROTECT 10-12	152^a^	18 (11.8)	<LOD	<LOD	<LOD	<LOD	0.6	0.9
			0.4	NHANES 07-08	364	121 (33.2)	<LOD	<LOD	<LOD	0.5	1.1	8.3
	2,4,5-T	2,4,5-T	0.1	PROTECT 10-12	152^a^	0 (0.0)	<LOD	<LOD	<LOD	<LOD	<LOD	<LOD
			0.1	NHANES 07-08	364	3 (0.8)	<LOD	<LOD	<LOD	<LOD	<LOD	1.2

*Abbreviations:* *GM* geometric mean, *LOD* limit of detection, *NHANES* National Health and Nutrition Examination Survey, *PROTECT* Puerto Rico Testsite for Exploring Contamination Threats. ^a^152 samples from 54 women; ^b^116 samples from 54 women; ^c^141 samples from 54 women.

**Table 2:** **Associations between time of urine collection or demographic characteristics and urinary concentrations of pesticide biomarkers**

Variable	DCBA^a^		DHMB		3-PBA		***trans***-DCCA		2,4-D	
	N	β (95% CI)^b^	N (≥LOD)	OR (95% CI)^c^	N (≥LOD)	OR (95% CI)^c^	N (≥LOD)	OR (95% CI)^c^	N (≥LOD)	OR (95% CI)^c^
**Time of day**										
PM	65	0.0 (-0.4, 0.4)	67 (18)	1.4 (0.7, 2.8)	61 (29)	1.1 (0.5, 2.2)	67 (5)	1.1 (0.3, 4.0)	67 (11)	2.2 (0.8, 6.1)
AM	82		85 (18)	1.0	80 (36)	1.0	85 (6)	1.0	85 (7)	1.0
**Age (years)**										
<24	76	0.3 (-0.4, 1.0)	80 (18)	0.9 (0.4, 2.0)	75 (36)	1.2 (0.6, 2.4)	80 (8)	2.6 (0.7, 9.8)	80 (11)	1.5 (0.4, 5.2)
≥24	71		72 (18)	1.0	66 (29)	1.0	72 (3)	1.0	72 (7)	1.0
**Educ. (years)**										
≤12	33	0.9 (0.1, 1.7)*	36 (11)	1.6 (0.6, 4.2)	32 (16)	1.2 (0.5, 3.0)	36 (6)	4.4 (1.3, 15.6)*	36 (2)	0.4 (0.1, 1.6)
>12	114		116 (25)	1.0	109 (49)	1.0	116 (5)	1.0	116 (16)	1.0
**Married**										
Yes	67	0.0 (-0.4, 0.4)	68 (12)	0.5 (0.2, 1.2)	65 (33)	1.5 (0.7, 3.1)	68 (4)	0.8 (0.2, 2.9)	68(13)	3.5 (1.5, 8.4)*
No	79		80 (23)	1.0	73 (30)	1.0	80 (6)	1.0	80 (5)	1.0
**Unemployed**										
Yes	43	-0.5 (-1.2, 0.2)	43 (16)	2.7 (1.1, 6.3)*	36 (15)	0.8 (0.4, 1.8)	43 (3)	1.1 (0.3, 4.3)	43 (5)	0.9 (0.3, 3.1)
No	103		105 (19)	1.0	102 (48)	1.0	105 (7)	1.0	105 (13)	1.0

*Abbreviations:* *β* beta coefficient, *CI* confidence interval, *LOD* limit of detection, *OR* odds ratio.

^a^Biomarker concentration was sg-corrected and log-transformed; ^b^β and associated 95% CI calculated using a linear mixed model with a random subject effect and a fixed effect for time of urine collection or demographic characteristic; ^c^OR and 95% CI calculated using a generalized estimating equation with a fixed effect for time of urine collection or demographic characteristic; *p <0.05.

**Table 3:** **Associations between select food items consumed in the past 48-hr and urinary concentrations of pesticide biomarkers**

Variable	DCBA^a^		DHMB		3-PBA		***trans***-DCCA		2,4-D	
	N	β (95% CI)^b^	N (≥LOD)	OR (95% CI)^c^	N (≥LOD)	OR (95% CI)^c^	N (≥LOD)	OR (95% CI)^c^	N (≥LOD)	OR (95% CI)^c^
**Apples**										
Yes	27	0.1 (-0.5, 0.6)	28 (8)	1.4 (0.6, 3.3)	26 (11)	0.9 (0.4, 2.0)	28 (2)	1.2 (0.3, 5.5)	28 (3)	0.9 (0.3, 2.7)
No	113		114 (25)	1.0	106 (49)	1.0	114 (7)	1.0	114 (13)	1.0
**Cherries**										
Yes	8	0.3 (-0.6, 1.2)	9 (2)	0.9 (0.2, 4.3)	8 (1)	0.2 (0.0, 1.5)	9 (1)	2.0 (0.2, 19.2)	9 (1)	1.0 (0.1, 7.0)
No	132		133 (31)	1.0	124 (59)	1.0	133 (8)	1.0	133 (15)	1.0
**Collards**										
Yes	5	-0.2 (-1.4, 0.9)	5 (1)	0.8 (0.1, 7.9)	5 (3)	1.8 (0.2, 14.5)	5 (0)	—	5 (2)	5.9 (1.3, 26.7)*
No	135		137 (32)	1.0	127 (57)	1.0	137 (9)	1.0	137 (14)	1.0
**Grapes**										
Yes	48	-0.1 (-0.5, 0.4)	49 (9)	0.6 (0.3, 1.6)	45 (16)	0.5 (0.2, 1.2)	49 (2)	0.5 (0.1, 2.5)	49 (6)	1.2 (0.3, 3.8)
No	92		93 (24)	1.0	87 (44)	1.0	93 (7)	1.0	93 (10)	1.0
**Grape juice**										
Yes	55	-0.4 (-0.8, 0.1)	55 (9)	0.5 (0.2, 1.2)	49 (20)	0.7 (0.4, 1.4)	55 (4)	1.3 (0.3, 5.5)	55 (6)	0.9 (0.4, 2.3)
No	85		87 (24)	1.0	83 (40)	1.0	87 (5)	1.0	87 (10)	1.0
**Peanuts**										
Yes	21	-0.4 (-0.9, 0.1)	22 (3)	0.5 (0.1, 2.3)	20 (7)	0.6 (0.2, 1.6)	22 (2)	1.6 (0.3, 9.4)	22 (2)	0.8 (0.2, 2.9)
No	119		120 (30)	1.0	112 (53)	1.0	120 (7)	1.0	120 (14)	1.0
**Peanut butter**										
Yes	14	-0.4 (-1.1, 0.3)	14 (4)	1.4 (0.5, 3.9)	13 (6)	1.0 (0.3, 3.3)	14 (2)	2.9 (0.6, 15.1)	14 (1)	0.6 (0.1, 5.1)
No	126		128 (29)	1.0	119 (54)	1.0	128 (7)	1.0	128 (15)	1.0
**Raisins**										
Yes	15	-0.4 (-1.1, 0.3)	16 (2)	0.4 (0.1, 2.0)	15 (6)	0.8 (0.4, 1.6)	16 (1)	1.0 (0.1, 8.8)	16 (1)	0.5 (0.1, 4.4)
No	125		126 (31)	1.0	117 (54)	1.0	126 (8)	1.0	126 (15)	1.0
**Spinach**										
Yes	6	-0.6 (-1.6, 0.3)	6 (0)	—	6 (3)	1.2 (0.2, 8.1)	6 (1)	3.2 (0.3, 33.2)	6 (2)	4.4 (1.1, 17.9)*
No	134		136 (33)	1.0	126 (57)	1.0	136 (8)	1.0	136 (14)	1.0
**Strawberries**										
Yes	33	-0.1 (-0.6, 0.4)	34 (8)	1.0 (0.4, 2.5)	34 (12)	0.6 (0.3, 1.3)	34 (3)	1.6 (0.5, 5.7)	34 (3)	0.7 (0.2, 2.8)
No	107		108 (25)	1.0	98 (48)	1.0	108 (6)	1.0	108 (13)	1.0
**Tomatoes**										
Yes	59	0.1 (-0.3, 0.5)	60 (13)	0.9 (0.4, 1.8)	56 (25)	0.9 (0.5, 1.9)	60 (6)	2.9 (0.7, 13.2)	60 (5)	0.6 (0.2, 1.8)
No	81		82 (20)	1.0	76 (35)	1.0	82 (3)	1.0	82 (11)	1.0

*Abbreviations:* *β* beta coefficient, *CI* confidence interval, *LOD* limit of detection, *OR* odds ratio.

^a^Biomarker concentration was sg-corrected and log-transformed; ^b^β and associated 95% CI calculated using a linear mixed model with a random subject effect and a fixed effect for food consumed in the past 48-hr; ^c^OR and 95% CI calculated using a generalized estimating equation with a fixed effect for food consumed in the past 48-hr; *p <0.05.

**Table 4:** **Associations between pest-related issues and urinary concentrations of pesticide biomarkers**

Variable	DCBA^a^		DHMB		3-PBA		***trans***-DCCA		2,4-D	
	N	β (95% CI)^b^	N (≥LOD)	OR (95% CI)^c^	N (≥LOD)	OR (95% CI)^c^	N (≥LOD)	OR (95% CI)^c^	N (≥LOD)	OR (95% CI)^c^
**Insects (inside)** ^**d**^										
Yes	71	0.2 (-0.2, 0.7)	73 (20)	1.6 (0.7, 3.9)	69 (35)	1.6 (0.8, 3.2)	73 (9)	—	73 (7)	0.7 (0.2, 2.2)
No	69		69 (13)	1.0	63 (25)	1.0	69 (0)	1.0	69 (9)	1.0
**Pesticides (inside)** ^**e**^										
Yes	6	-0.2 (-1.2, 0.8)	7 (1)	0.5 (0.1, 5.0)	6 (1)	0.2 (0.0, 2.1)	7 (1)	2.6 (0.3, 26.2)	7 (0)	—
No	134		135 (32)	1.0	126 (59)	1.0	135 (8)	1.0	135 (16)	1.0
**Pesticides (inside)** ^**f**^										
Yes	31	0.3 (-0.2, 0.7)	32 (9)	1.4 (0.5, 3.6)	29 (15)	1.4 (0.6, 3.4)	32 (5)	4.9 (1.1, 22.1)*	32 (2)	0.5 (0.1, 2.2)
No	109		110 (24)	1.0	103 (45)	1.0	110 (4)	1.0	110 (14)	1.0
**Pesticides (outside)** ^**g**^										
Yes	11	0.3 (-0.4, 1.0)	11 (2)	0.7 (0.1, 3.6)	11 (5)	1.0 (0.3, 3.3)	11 (1)	1.5 (0.2, 13.8)	11 (1)	0.8 (0.1, 6.7)
No	128		130 (31)	1.0	120 (55)	1.0	130 (8)	1.0	130 (15)	1.0
**Pesticides (home/lawn)** ^**h**^										
Yes	16	0.6 (-0.0, 1.2)	17 (7)	2.7 (0.9, 8.0)	17 (10)	1.9 (0.6, 5.9)	17 (3)	4.3 (0.7, 24.7)	17 (1)	0.5 (0.1, 4.0)
No	124		125 (26)	1.0	115 (50)	1.0	125 (6)	1.0	125 (15)	1.0
**Pesticides(stored)** ^**i**^										
Yes	95	-0.4 (-0.9, 0.0)	97 (20)	0.6 (0.3, 1.4)	92 (41)	0.9 (0.4, 2.0)	97 (7)	1.7 (0.3, 9.1)	97 (8)	0.4 (0.1, 1.5)
No	45		45 (13)	1.0	40 (19)	1.0	45 (2)	1.0	45 (8)	1.0
**Insect repellent** ^**j**^										
Yes	16	1.2 (0.4, 2.0)*	17 (6)	0.3 (0.1, 1.3)	17 (8)	0.7 (0.2, 2.3)	17 (3)	0.1 (0.0, 1.5)	17 (3)	0.8 (0.1, 5.0)
No	33		33 (5)	1.0	29 (11)	1.0	33 (1)	1.0	33 (2)	1.0

*Abbreviations:* *β* beta coefficient, *CI* confidence interval, *LOD* limit of detection *OR* odds ratio.

^a^Biomarker concentration was sg-corrected and log-transformed; ^b^β and associated 95% CI calculated using a linear mixed model with a random subject effect and a fixed effect for pest-related issue; ^c^OR and 95% CI calculated using a generalized estimating equation with a fixed effect for home pest-related issue; ^d^insects a common nuisance inside home; ^e^pesticides applied inside home by a professional exterminator regardless of time since last application; ^f^pesticides applied inside home by participant regardless of time since last application; ^g^pesticides applied outside home by participant regardless of time since last application; ^h^pesticides applied to home or lawn by participant in past 48-hr; ^i^pesticides currently stored inside home; ^j^insect repellent spray, lotion, or towelette use since becoming pregnant (information was collected at visit 2 only, thus effect estimates were derived from linear or logistic regression models); *p <0.05.

### Statistical analysis

Statistical analysis was performed using SAS version 9.3 for Windows (SAS Institute, Cary, NC, USA). Distributions of urinary concentrations were calculated and compared to those measured in U.S. women 18–40 years of age from NHANES where available (http://www.cdc.gov/nchs/nhanes.htm). In further statistical analyses, we excluded biomarkers that were detected in less than 5% of the samples (DEET, 4-F-3-PBA, *cis*-DBCA, and 2,4,5-T). To assess between- and within-subject variability in urinary concentrations over the three study visits, intraclass correlation coefficients (ICCs) were calculated using variance components derived from linear mixed models with a random subject effect only for log-transformed analyte concentrations detected in at least 50% of the samples (DCBA only). The corresponding 95% confidence intervals (CIs) associated with the ICCs were also calculated [42]. The magnitude of the ICCs was interpreted using the following criteria: poor reproducibility (ICC <0.40), fair to good reproducibility (0.40 ≤ ICC <0.75), and excellent reproducibility (ICC ≥0.75) [43]. To assess whether or not there are trends in urinary concentrations over pregnancy, we also ran a Friedman test, a non-parametric equivalent of the repeated measures analysis of variance test, for analytes detected in at least 50% of the samples. To identify predictors of pesticide exposure, we also examined the associations between time of urine collection, demographic characteristics, select food items consumed in the past 48-hr, or pest-related issues and urinary concentrations of the analytes in one of two ways. For biomarkers detected in less than 50% of the samples (DHMB, 3-PBA, *trans*-DCCA, and 2,4-D), we estimated the odds of having a detectable biomarker concentration given a particular variable (e.g., consumed apples in the past 48-hr) relative to the odds of the outcome in the absence of that variable (e.g., did not consume apples in the past 48-hr). In other words, these statistical models relied on binary exposure data that assigned a participant a “yes” if the biomarker was detected or a “no” if the biomarker was not detected. We reported the associations as odds ratios (ORs) along with their associated 95% CIs, which were calculated using generalized estimating equations to account for repeated measures with a fixed effect for the predictor of interest. In this case, we gave consideration to modeling urinary biomarker concentrations as a continuous variable, but we chose a binary outcome approach (i.e., detect or non-detect) as the former would require the imputation of too many left censored values for many of the biomarkers (e.g., 92.8% of values for *trans*-DCCA) and, as a result, may not be the most valid statistical approach. On the other hand, for analytes detected in at least 50% of the samples, we modeled urinary biomarker concentrations as a log-transformed continuous variable and reported the beta coefficients along with their associated 95% CIs, which were calculated using linear mixed effect models to account for repeated measures with a random subject effect and a fixed effect for the predictor of interest. Because information on insect repellent use was only collected during visit 2, statistical models accounting for repeated measurements were not necessary and, as a result, associations with visit 2 biomarker concentrations were assessed using either logistic or linear regression models for that variable. We only assessed those questionnaire items with N ≥5 in the “Yes” and “No” groups. Finally, for exposure biomarkers with multiple significant predictors, to explore confounding we constructed multivariable models where these predictors were included simultaneously.

## Results

Table 1 shows the distributions of the urinary biomarkers relative to those in U.S. women ages 18–40 from NHANES. In this sample of pregnant Puerto Rican women, 152 urine samples were available for analysis for most analytes, except for 4-F-3-PBA (N = 116) and 3-PBA (N = 141) due to quality control issues and the presence of interfering compound(s) in the urine, respectively. All analytes were detected in fewer than 50% of the samples, except DCBA, which was detected in 79%. Ninety-fifth percentile urinary concentrations of DEET (<LOD = 0.1 ng/ml vs. 0.2 ng/ml), 3-PBA (2.3 ng/ml vs. 6.5 ng/ml), *trans*-DCCA (2.4 ng/ml vs. 3.3 ng/ml), and 2,4-D (0.6 ng/ml vs. 1.1 ng/ml) were lower than the U.S. population-based sample, whereas 95th percentile urinary concentrations of 4-F-3-PBA (both < LOD = 0.1 ng/ml), *cis*-DBCA (both < LOD = 0.5 ng/ml), and 2,4,5-T (both < LOD = 0.1 ng/ml) were comparable in both cohorts.The ICCs for urinary concentrations of DCBA uncorrected and corrected for SG were 0.60 (95% CI: 0.45, 0.74) and 0.60 (95% CI: 0.44, 0.73), respectively, demonstrating fair to good reproducibility. As shown in Figure 1, the distributions of urinary levels of DCBA were also not significantly different between the three time points measured. No ICCs or Friedman tests were calculated for the other analytes because detection frequencies were <50%.

Table 2 shows the associations between time of urine collection or demographic characteristics and urinary concentrations of pesticide biomarkers. There were no statistically significant associations between urinary concentrations of the analytes and time of urine collection (AM vs. PM) or woman’s age (<24 vs. ≥24 years). However, less educated women (≤12 years of education) were more likely to have greater urinary concentrations of DCBA (β: 0.9, 95% CI: 0.1, 1.7; geometric means of less educated vs. more educated women: 8.0 ng/ml vs. 3.4 ng/ml) and *trans*-DCCA (OR: 4.4, 95% CI: 1.3, 15.6) compared with more educated women. Married women were more likely to have detectable urinary concentrations of 2,4-D (OR: 3.5, 95% CI: 1.5, 8.4) relative to unmarried women. Unemployed women were also more likely to have detectable urinary concentrations of DHMB (OR: 2.7, 95% CI: 1.1, 6.3) compared to employed women.

Shown in Table 3 are the associations between the consumption of select fruits, vegetables, or legumes in the past 48-hr and urinary concentrations of pesticide biomarkers. We found no statistically significant associations between urinary concentrations of the biomarkers and consumption of nearly all fruits, vegetables, and legumes. However, women that consumed collards (OR: 5.9, 95% CI: 1.3, 26.7) or spinach (OR: 4.4, 95% CI: 1.1, 17.9) in the past 48-hr were more likely to have detectable urinary concentrations of 2,4-D relative to women that did not consume those foods in the past 48-hr. Associations between urinary concentrations of the pesticides biomarkers and the participants’ consumption of other foods (Brussels sprouts, celery, or wine) in the past 48-hr were not assessed due to the low number of women who reported consuming those items.

Table 4 shows the associations between pest-related issues and urinary concentrations of pesticide biomarkers. There were no statistically significant associations if insects were a common nuisance in the home, pesticides had been applied outside the home by the participant or inside the home by a professional exterminator (regardless of time since last application), or pesticides were currently stored in the home. However, if the participant applied pesticides inside the home (regardless of time since last application), there was significant increase in the odds of having a detectable urinary concentration of *trans*-DCCA (OR: 4.9, 95% CI: 1.1, 22.1) compared to other women. In addition, insect repellent use since becoming pregnant was positively associated with urinary concentrations of DCBA (β: 1.2, 95% CI: 0.4, 2.0; geometric means of users vs. non-users: 8.3 ng/ml vs. 2.7 ng/ml). Associations between urinary concentrations of the pesticides biomarkers and the participants’ use of pet grooming products, pet flea/tick prevention applications, or pet flea/tick spray in the past 48-hr were not assessed due to the low number of women who reported use of those items.

Finally, the predictors that were statistically significant were simultaneously included in multivariate models for each biomarker that had more than one statistically significant predictor (e.g., 2,4-D in relation to both marital status and spinach or collards consumption). While this served to increase p-values somewhat due to reduced statistical power, effect estimates were similar (results not shown).

## Discussion

To our knowledge, this is the first biomarker study to report on exposures to select pesticides among pregnant women in Puerto Rico, and also the first to report on temporal variability of DCBA, an oxidative metabolite of DEET. We found that 95th percentile urinary concentrations of DEET, 3-PBA, *trans*-DCCA, and 2,4-D were lower than urinary concentrations in women of reproductive age on the mainland U.S., whereas 95th percentile urinary concentrations of 4-F-3-PBA, *cis*-DBCA, and 2,4,5-T were similar in both populations. We also found evidence that demographic factors, consumption of leafy greens, and pesticide use are potentially important determinants of exposure to certain pesticides among this group of pregnant women.

Aside from NHANES, there have been only a few studies in other pregnancy cohorts from around the world that have reported on urinary concentrations for some of these analytes [11–13, 30, 32, 44]. Perhaps most relevant to our findings, in a study of pyrethroid exposures among pregnant women from 10 Caribbean countries (the authors did not include the Commonwealth of Puerto Rico), urinary detection frequencies for data across all countries were between 77-100% for *cis*-DBCA, *trans*-DCCA, 3-PBA, and 4-F-3-PBA [12]. In our study, detection frequencies were between 0–46.1% for the same group of metabolites. However, the LODs in the Caribbean study were about 1–2 orders of magnitude lower than those in our study, and, as a result, the greater detection frequency in the Caribbean study may not necessarily be equivalent to greater exposure. For example, it is notable that the 95th percentile urinary concentration of 3-PBA was 2.3 ng/ml in our study, compared to 0.54 ng/ml in the Caribbean study, which suggests that exposure to some pyrethroids may have been greater in our population.

The temporal reliability analysis for DCBA suggests that more than one spot sample may be needed to characterize exposure to DEET in pregnant women over the course of pregnancy. Thus, epidemiology studies conducted in pregnant women where the exposure assessment strategy relies on DCBA concentrations from a single spot sample will likely result in a moderate level of exposure misclassification, which, if non-differential, would underestimate (bias toward the null) true associations. In other published studies, temporal variability of urinary concentrations of the pesticides and pesticide metabolites assessed in our study has largely been unexplored, especially over a time period of several months which would be most relevant to pregnancy. ICCs for creatinine-corrected 3-PBA (0.85) and SG-corrected 2,4-D (0.57) associated with spot urine samples collected in Polish adults over seven consecutive days [45] and up to six spot urine samples collected in U.S. adults over a 48-hr period [46], respectively, have been reported.

We found that pregnant women were more likely to have detectable urinary concentrations of DHMB if they were unemployed. One hypothesis explaining this finding is that unemployed women may spend more time outdoors (e.g., gardening, exercising), thus necessitating increased use of insect repellents containing DEET, relative to working women. We had questionnaire information on weekly exercise and chore frequency and duration, which may correlate with outdoor activities, but further investigation of these variables with respect to employment status did not reveal any notable relationships (data not shown).

Participants in our study were also more likely to have detectable urinary concentrations of the herbicide 2,4-D if they were married. One plausible explanation is that married women might be more likely to be home owners relative to unmarried women and, as a result, might be more likely to be involved with home upkeep activities, such as controlling weed growth with herbicides containing 2,4-D. Unfortunately, information on home ownership was not collected so this hypothesis could not be explored further. Positive relationships between being married or living in an owned residence and urinary concentrations of 3-PBA, a non-specific metabolite of several pyrethroid insecticides, among U.S. pregnant women have been reported [30].

In addition, less educated women in our study were more likely to have greater urinary concentrations DCBA and *trans*-DCCA than other women, a finding supported by other studies for *trans*-DCCA [13], but not 3-PBA [30]. One hypothesis is that compared with more educated women, less educated women had lower household incomes, which resulted in lifestyle patterns (e.g., insufficient income to purchase organic produce) and/or conditions (e.g., living in poorer communities with decreased access to organic produce) with increased risk of exposure. For example, in a study of U.S. adults [47], consumption of organic produce was shown to increase with both educational attainment and household income levels, which supports our hypothesis.

Furthermore, we found a positive association between participants’ use of pesticides at home and urinary concentration of *trans*-DCCA. Similar findings have been observed with pyrethroid metabolites in pregnant Chinese women [13], but not in Italian adults [31]. Use of insect repellents since becoming pregnant was also a positive predictor of urinary concentrations of DCBA, which is a novel finding that may validate DCBA as a biomarker of DEET exposure.

Participants in our study were also more likely to have detectable urinary concentrations of 2,4-D if they consumed collards or spinach in the past 48-hr. Consumption of unspecified “leafy greens” [31] or unspecified “dark green vegetables” [10] has also been reported in adults to be positively associated with urinary concentrations of 3-PBA. Consistent with our analysis, no associations between age and urinary concentrations of 3-PBA and *trans*-DCCA have been reported by others [10, 13, 30, 31].

Several strengths of our study include focusing on an understudied and potentially at-risk population, evaluating biomarkers of understudied exposures, and the collection of repeated data, which allowed for a powerful analysis where each participant served as her own reference in longitudinal models. One primary limitation was the lack of detailed questionnaire information, especially information on how demographic characteristics and pesticide use may lead to increased exposures. Although information of this kind would have assisted with understanding our findings, the increase in detail on the questionnaires would have increased participant burden and may have resulted in reduced participation and study compliance or potentially introduced added recall error. The interpretation of our findings was also further complicated by limited publicly-available information on pesticide use patterns in Puerto Rico. Additional limitations which precluded further quantitative analyses included the modest sample size (N = 54), the small number of participants reporting consumption of certain food items or performing certain pest-related activities, and the relatively low detection frequencies (<50%) of most urinary biomarkers. Finally, it should be noted that the results of our study may not be generalizable to other populations, especially of young children, because interaction with sources of pesticides and/or their metabolism of pesticides may be different from those of pregnant women or other adults.

## Conclusions

We showed that pesticide use in a group of Puerto Rican pregnant women is associated with exposure to several pesticides. We also showed that when using DCBA as a specific urinary biomarker of DEET, if possible, more than one sample should be collected over the course of the pregnancy to minimize exposure measurement error. We demonstrated that among these Puerto Rican pregnant women being married, not employed, or less educated was associated with certain biomarkers of exposure to pesticides and should be further investigated to understand what aspects of these demographic characteristics lead to greater exposures. Consumption of collards, spinach, and potentially other leafy greens may also be important determinants of exposure to certain pesticides. Because human exposure to pesticides may be associated with adverse health effects, further research will increase our understanding of the drivers of pesticide exposure among pregnant women.

## Abbreviations

CDC: Centers for disease control and prevention
CI: Confidence interval
2: 4-D: 2,4-dichlorophenoxyacetic acid
DBCA: cis-3-(2,2-dibromovinyl)-2,2-dimethylcyclopropane carboxylic acid
DCBA: 2-diethyl-carbamoyl benzoic acid
DCCA: Trans-3-(2,2-dichlorovinyl)-2,2-dimethylcyclopropane carboxylic acid
DEET: N-N-diethyl-meta-toluamide
DHMB: N,N-diethyl-3-hydroxymethylbenzamide
4-F-3-PBA: 4-fluoro-3-phenoxybenzoic acid
ICC: Intraclass correlation coefficient
LOD: Limit of detection
NHANES: National health and nutrition examination survey
NIH: National institutes of health
OR: Odds ratio
3-PBA: 3-phenoxybenzoic acid
PROTECT: Puerto Rico testsite for exploring contamination threats
SG: Specific gravity
2: 4,5-T: 2,4,5-trichlorophenoxyacetic acid.
